# The Role of Endoscopic Cyclophotocoagulation as a Primary Alternative Treatment for Patients at Risk of Ocular Hypotony: A Case Report

**DOI:** 10.7759/cureus.78204

**Published:** 2025-01-29

**Authors:** Akshatha Daniel, Minak Bhalla

**Affiliations:** 1 Hospital Medicine, Chelsea and Westminster Hospital NHS Foundation Trust, London, GBR; 2 Ophthalmology, Royal Free Hospital, London, GBR

**Keywords:** covid-19 complications, endoscopic cyclophotocoagulation (ecp), glaucoma, ocular hypotony, preserflo

## Abstract

Endoscopic cyclophotocoagulation (ECP) offers a viable alternative for managing advanced primary open-angle glaucoma (POAG) in patients at risk of ocular hypotony. We describe a case of a successful outcome with ECP in a patient who developed ocular hypotony secondary to Preserflo surgery. A 93-year-old South Asian male experienced significant visual field deterioration and ocular hypotony following Preserflo surgery on the left eye, complicated by a severe cough from COVID-19. ECP was successfully utilized in the at-risk fellow eye, demonstrating stable intraocular pressures with reduced dependency on glaucoma medications over a 10-month follow-up period. Our findings suggest that ECP may be a preferable initial approach in managing POAG patients at elevated risk of hypotony or non-attendance, offering a safer alternative with comparable efficacy to traditional filtration surgeries. This case highlights the importance of selecting appropriate glaucoma interventions based on individual risk profiles. It underscores the necessity of strict postoperative follow-ups, particularly in the context of potential complications from systemic illnesses like COVID-19.

## Introduction

Ocular hypotony is a recognized complication of glaucoma surgery, with severe cases potentially leading to vision loss and choroidal effusions. In individuals with advanced primary open-angle glaucoma (POAG), glaucoma filtration surgery is often required to achieve target intraocular pressure (IOP) [1]. However, attaining this target pressure from the outset can be challenging, as overfiltration, ciliary body shutdown, or unintentional leakage may lead to reduced IOP, resulting in ocular hypotony [2].

Endoscopic cyclophotocoagulation (ECP) is a technique in which ciliary processes are partially ablated to reduce the secretion of aqueous humor, thereby lowering IOP [3]. This procedure is commonly performed alongside cataract surgery. Studies have shown that patients experience significant reductions in IOP and dependence on eye drops over a three-year follow-up period [3]. Although ocular hypotony is a potential complication, its incidence is significantly lower than in glaucoma filtration surgeries [4].

Preserflo is a form of microinvasive glaucoma filtration surgery. It consists of a biocompatible tube implanted in the eye to reduce IOP by creating a new drainage pathway [5]. Common postoperative adverse events include ocular hypotony, device contact with the iris, and a shallow or flat anterior chamber [6].

We present a case report of ocular hypotony following Preserflo surgery in a patient who experienced severe coughing due to COVID-19. Additionally, we describe the successful outcome of ECP in the alternate eye to manage his POAG while avoiding the complication of ocular hypotony.

## Case presentation

**Figure 1:**
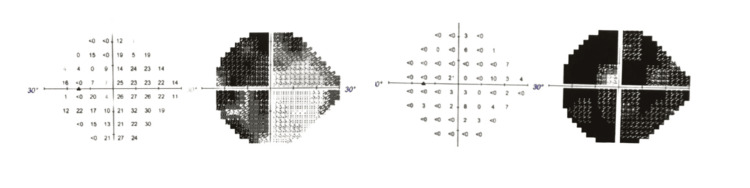
Left eye Humphrey central 24-2 visual field Visual fields taken one year apart showing worsening of vision despite being on multiple drops and stent insertion

A 93-year-old South Asian male presented to the glaucoma clinic for a postoperative evaluation four weeks after Preserflo surgery in his left eye. Previously, despite maximal medical management, including Trusopt, DuoTrav, Simbrinza, and Travaprost, his intraocular pressure reached up to 25 mmHg, as measured using Goldmann tonometry, and his visual field continued to deteriorate, as shown in the Humphrey visual field (Figure 1). This may have been partly due to compliance issues caused by multiple drop allergies, such as pain and excessive eye watering. He was listed for phacoemulsification and intraocular lens insertion, including iStent insertion, and was discharged with Simbrinza eye drops. Unfortunately, he was noncompliant with the Simbrinza drops as they caused him pain, and adequate target pressure could not be achieved three months after cataract surgery combined with iStent. A case-based approach, considering the patient’s best interests, was taken to proceed with the insertion of the Preserflo.

The surgery was successfully performed, and the patient was reviewed as per protocol post-operation. On postoperative day 1, his intraocular pressure was 3 mmHg without any sequelae of hypotony. Unfortunately, he failed to attend his follow-up appointment between day 1 and day 28 due to illness from COVID-19. At the four-week postoperative visit, he complained of worsening vision over a two-week period. He also admitted that during his illness with COVID-19, he experienced severe bouts of coughing and vomiting.

Examination of the left eye showed a large, diffuse, well-draining bleb. The Preserflo was observed in position in the anterior chamber. There was no bleb leak on fluorescein staining. However, the anterior chamber was shallow, and examination of the posterior chamber revealed large kissing choroidals, which were confirmed on B-scan.

Based on the above findings, a diagnosis of ocular hypotony secondary to an overfiltering bleb was made. Symptoms of his COVID-19 infection likely exacerbated this condition. Following this, he was treated with an orbital floor steroid, preservative-free dexamethasone eye drops, and atropine eye drops. In the interim, the vitreoretinal team was consulted, and they advised a watch-and-wait approach.

During the four-week follow-up, his left eye fundus examination showed improving choroidals. Examination of his fellow right eye revealed a high IOP of 26 mmHg. Given the patient's significant drop allergies and compliance issues, a decision was made to perform ECP instead of commencing new eye drops. Filtration surgery was also avoided at this stage. This decision was partly influenced by the emergence of new COVID-19 variants and the patient's age, as he remained at high risk for hypotony. He was scheduled to undergo the procedure in two weeks.

ECP involves direct visualization of the ciliary processes and is a minimally invasive procedure. The energy delivery, ranging from 100-300 mW, was applied in a continuous wave, sufficient to achieve the desired effect on the tissues. The procedure was successfully performed without complications.

**Figure 2:**
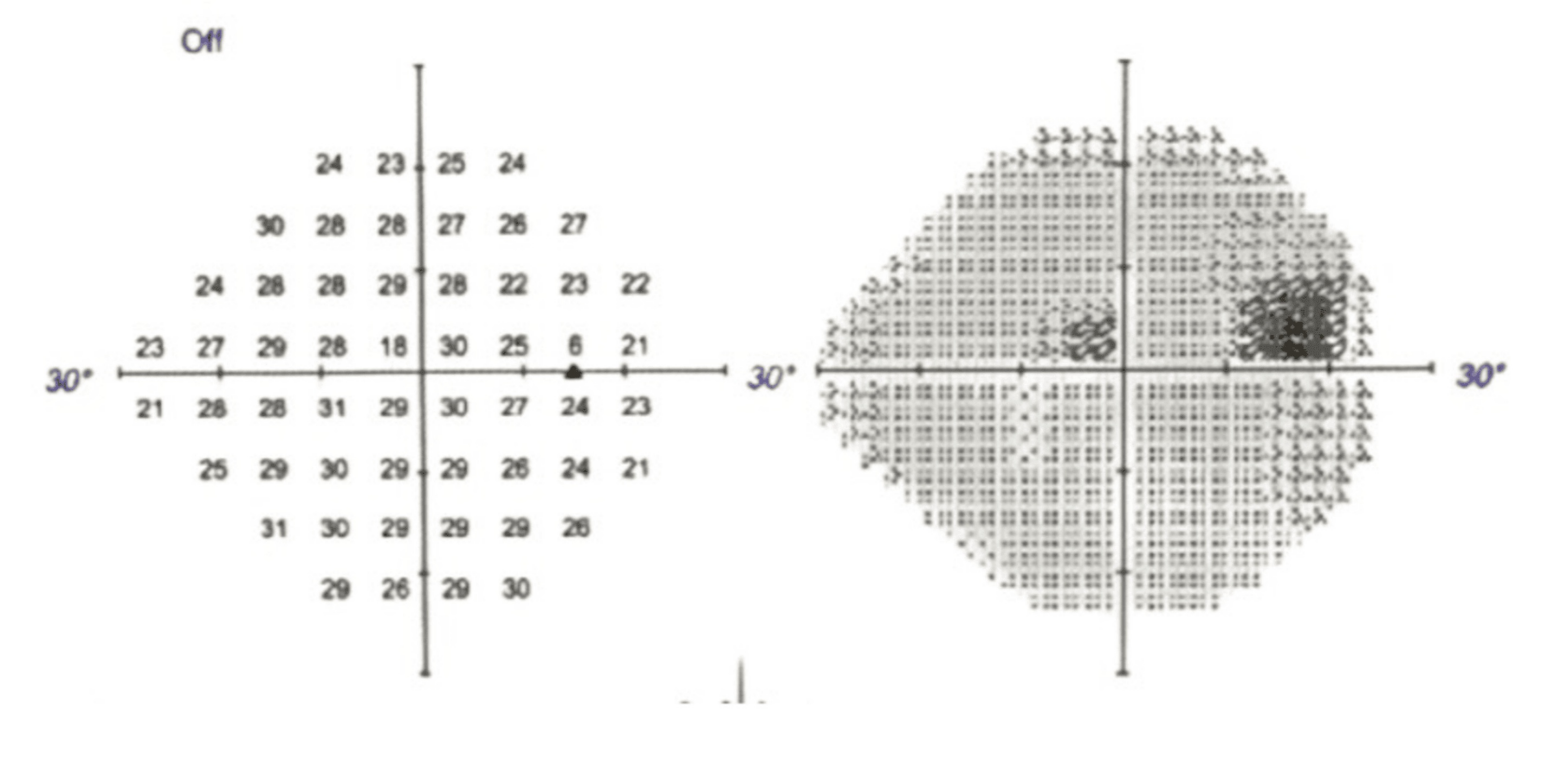
Right eye Humphrey central 24-2 visual field post-ECP Visual field of the right eye taken two months post-ECP showing preserved fields ECP: endoscopic cyclophotocoagulation

At his two-month postoperative follow-up, he was off all glaucoma eye drop medications, with both eyes showing stable pressures and vision in the right eye preserved (Figure 2). At the six-month post-ECP follow-up, his IOP measured 13 mmHg; at the 10-month mark, it was 16 mmHg. The patient was satisfied with the outcome of his right eye post-ECP, particularly as he no longer required glaucoma eye drops or filtration surgery.

## Discussion

**Figure 3:**
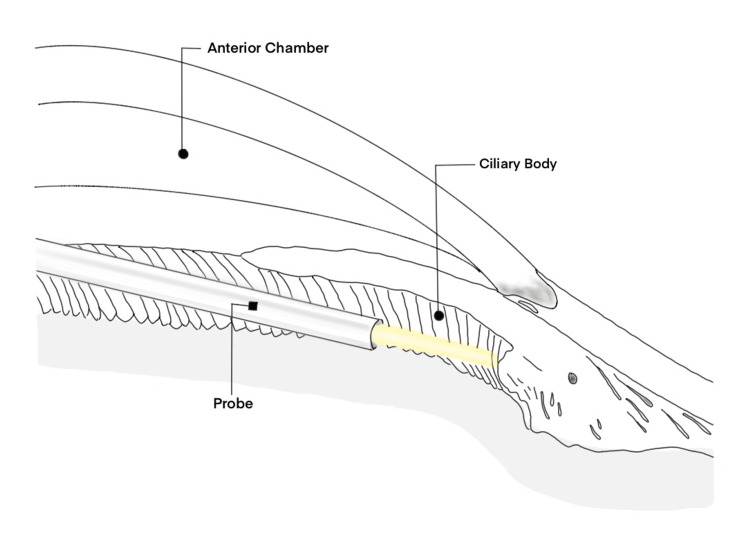
Mechanism of action of ECP Illustration showing ECP probe ablating ciliary body to reduce aqueous secretion ECP: endoscopic cyclophotocoagulation Image Credit: Author

In theory, Preserflo surgery and ECP increase the risk of hypotony through predominantly different mechanisms. ECP exclusively targets the secretion of aqueous humor (Figure 3). Thus, the complication of ocular hypotony following ECP is independent of overfiltration of aqueous humor and is not affected by variables that may influence this, unlike filtration surgeries [7]. One variable includes Valsalva-like activities, such as coughing and straining [8].

In our patient, along with the overfiltering bleb, observable factors contributing to low intraocular pressure include Valsalva-like activities such as coughing due to COVID-19 infection. These activities cause a transient rise in episcleral venous pressure, resulting in corresponding increases in intraocular pressure. This increase may lead to greater drainage of aqueous humor [8]. The nature of his cough resulted in over-drainage, leading to hypotony and vision deterioration. ECP was performed as the primary procedure, as it targets the ciliary production of aqueous humor and is less likely to be affected by the patient’s coughing.

Following risk stratification, an alternative approach could be to stent the Preserflo with 9-0 nylon and remove it once sufficient healing has occurred to reduce the risk of overfiltration [9].

A study that compared the safety and efficacy of ECP versus trabeculectomy (a bleb-forming filtration surgery similar to Preserflo surgery) suggested that ECP is safer than filtration surgeries. Additionally, the difference in mean intraocular pressure at six months post-procedure was not statistically significant. However, it should be noted that patients treated with ECP may have a higher incidence of requiring postoperative medications [4].

An alternate mode of cyclophotocoagulation, known as micropulse transscleral cyclophotocoagulation (MP-TSCPC), is often chosen for patients with refractory glaucoma, particularly in cases where previous surgical interventions have failed or for patients with poor visual potential. It is especially beneficial for those who require a less invasive procedure due to the risks associated with more traditional surgeries. Ideal candidates include patients with advanced glaucoma, where the preservation of minimal residual vision is critical, and those who may not tolerate traditional surgeries due to systemic health issues [10].

Studies indicate that MP-TSCPC effectively reduces IOP with fewer complications than continuous-wave cyclophotocoagulation. A study demonstrated that MP-TSCPC resulted in significant IOP reduction while maintaining a lower incidence of postoperative inflammation and other complications. Long-term results suggest that while repeat treatments may be necessary, complication rates remain low, making it a suitable option for managing difficult glaucoma cases [11].

Recent comparative studies provide insight into specific scenarios where one treatment may be preferred over another. A study compared MP-TSCPC and ECP in a cohort of mixed glaucoma types and found that while both procedures offer significant IOP reduction, MP-TSCPC may be more favorable for patients with refractory glaucoma due to its non-invasive nature and repeatability. While both procedures have relatively low complication rates, the choice may depend on patient-specific factors such as the presence of a natural lens, previous ocular surgeries, and the eye's overall health [12].

Table 1 shows the comparison of the characteristics of MP-TSCPC and ECP.

**Table 1:** Comparison of the characteristics of MP-TSCPC and ECP The table highlights each technique's distinct approaches, methodologies, and applications, providing a clear contrast between MP-TSCPC and ECP in terms of their practical use in clinical settings. MP-TSCPC: micropulse transscleral cyclophotocoagulation, ECP: endoscopic cyclophotocoagulation

Aspect	MP-TSCPC	ECP
Approach	Noninvasive, external	Minimally invasive, internal
Target	Ciliary body (via scleral application)	Direct visualization of ciliary processes
Energy delivery	Micropulse diode laser	Continuous diode laser
Safety profile	Lower risk of inflammation and pain	Visualization ensures precision and potentially lowers the risk of collateral damage
Repeatability	Highly repeatable due to minimal damage	Limited repeatability due to the invasive nature
Clinical cases	Refractory glaucoma, high hypotony risk	Advanced glaucoma, suitable for combined procedures with cataract surgery, challenging anatomical cases

MP-TSCPC and ECP significantly enhance the management of complex glaucoma cases by providing safer, less invasive alternatives to traditional surgical interventions. They are particularly valuable for patients at high risk of surgical complications or in cases where conventional surgery is not viable. The choice of procedure is context-specific, depending on local guidelines and clinical settings. Their continued development and integration into clinical practice promise to improve outcomes for a broad spectrum of glaucoma patients, addressing unmet glaucoma care and management needs.

We acknowledge the limitations of the outcomes discussed, as they are based on a single case study. Further large case series or randomized controlled trials are needed before the results can be generalized. However, this case study is expected to contribute to the growing body of successful outcomes following ECP procedures in patients at risk of ocular hypotony. We will continue to closely follow up with our patient at our clinic.

## Conclusions

Our case report highlights the use of ECP in glaucomatous eyes at risk of ocular hypotony. ECP is a good option for patients at risk of ocular hypotony related to Valsalva-like activities, such as coughing and straining, as seen with Preserflo surgery.

This case further emphasizes the importance of adhering to the postoperative follow-up schedule, which is paramount in detecting potential complications. It is crucial to consider the systemic well-being of the patient post-glaucoma surgery, and patients should be safety-netted accordingly. In the context of post-glaucoma surgery, such safety-netting involves providing clear instructions to patients, monitoring recovery, and identifying any signs of complications for early intervention. It may also be beneficial to recommend codeine linctus or another cough suppressant, as seen in cataract surgeries, for patients at risk of coughing or straining.
